# Review of Oxepine-Pyrimidinone-Ketopiperazine Type Nonribosomal Peptides

**DOI:** 10.3390/metabo10060246

**Published:** 2020-06-15

**Authors:** Yaojie Guo, Jens C. Frisvad, Thomas O. Larsen

**Affiliations:** Department of Biotechnology and Biomedicine, Technical University of Denmark, Søltofts Plads, Building 221, DK-2800 Kgs. Lyngby, Denmark; yaguo@dtu.dk (Y.G.); jcf@bio.dtu.dk (J.C.F.)

**Keywords:** oxepine, nonribosomal peptides, bioactivity, biosynthesis, fungi, *Aspergillus*

## Abstract

Recently, a rare class of nonribosomal peptides (NRPs) bearing a unique Oxepine-Pyrimidinone-Ketopiperazine (OPK) scaffold has been exclusively isolated from fungal sources. Based on the number of rings and conjugation systems on the backbone, it can be further categorized into three types A, B, and C. These compounds have been applied to various bioassays, and some have exhibited promising bioactivities like antifungal activity against phytopathogenic fungi and transcriptional activation on liver X receptor α. This review summarizes all the research related to natural OPK NRPs, including their biological sources, chemical structures, bioassays, as well as proposed biosynthetic mechanisms from 1988 to March 2020. The taxonomy of the fungal sources and chirality-related issues of these products are also discussed.

## 1. Introduction

Nonribosomal peptides (NRPs), mostly found in bacteria and fungi, are a class of peptidyl secondary metabolites biosynthesized by large modularly organized multienzyme complexes named nonribosomal peptide synthetases (NRPSs) [1]. These products are amongst the most structurally diverse secondary metabolites in nature; they exhibit a broad range of activities, which have been exploited in treatments such as the immunosuppressant cyclosporine A and the antibiotic daptomycin [2,3]. Due to their high importance, a lot of bioengineering studies have been carried out to elucidate their biosynthetic pathways, increase their yields, and generate novel homologs [4,5]. Within the recent decades, a rarely observed class of NRPs containing an Oxepine-Pyrimidinone-Ketopiperazine (OPK) scaffold comprising three amino acids, including one or two anthranilic acid(s), has emerged since the isolation of cinereain 32 years ago [6]. Interestingly, the structures of OPK NRPs are close to some quinazolinone alkaloids, specifically types Q12 to Q18 quinazolinones, such as fumiquinazolines and benzomalvins mostly produced by *Aspergillus* and *Penicillium* species as summarized in a recent review covering 157 compounds [7]. One major difference of the core skeleton between OPK NRPs and those specific quinazolinones is that OPK compounds bear a unique oxepine moiety instead of a phenyl group. Additionally, the OPK compounds were also described as diketopiperazine alkaloids [8,9,10]. However, they were not included in recent reviews on quinazolinones or diketopiperazines [7,11,12,13,14]. More attention should be paid to this class of compounds, considering their various bioactivities and intriguing structures, although some synthetic efforts have already been made [15,16]. To get a comprehensive perspective, here we review different aspects of these OPK NRPs, including their biological sources, structures, bioactivities, and proposed biosynthesis, for the first time.

## 2. Results

### 2.1. Biological Sources and Chemical Structures

**Figure 1:**
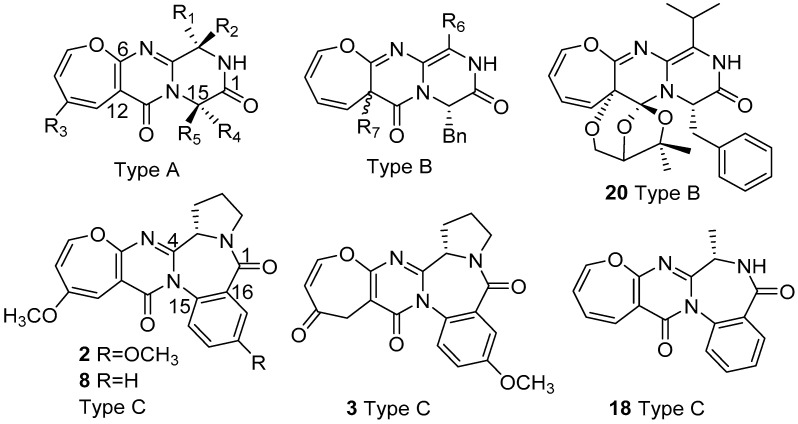
Structures of three types (A, B, and C) of Oxepine-Pyrimidinone-Ketopiperazine (OPK) nonribosomal peptides (NRPs).

Up to March 2020, thirty-five products bearing OPK backbone (Figure 1, Table 1, Table 2 and Table 3) have been isolated from natural sources, surprisingly all from fungi. The first compound reported was cinereain (**1**) from fungus *Botrytis cinerea* ATCC 64157 cultured on shredded wheat medium [6] followed by the isolation of asperloxin A (**2**) [17] and B (**3**) [18] from *Aspergillus ochraceus* DSM 7428, which was a part of One-Strain-Many-Compounds (OSMAC) approach to release the chemical diversity of this strain in A. Zeeck´s group [19]. Oxepinamides A–C (**4**–**6**) were reported to be isolated from the organic extract of the culture broth and mycelia of filamentous fungus *Acremonium* sp. grown in static liquid culture containing seawater-based medium [20]. Janoxepin (**7**) with a rare D-leucine residue was obtained from *Aspergillus janus* IBT 22274 cultivated on yeast extract sucrose (YES) medium [21]. Circumdatins A (**2**) and B (**8**), first reported to be benzodiazepines with two benzyl groups from *Aspergillus ochraceus* IBT 12704 as good chemotaxonomic markers [22], were later isolated from a marine-derived fungus *Aspergillus ostianus* strain 01F313, and their structures were revised to be oxepine-containing benzodiazepine alkaloids by X-ray crystallography [23]. The structure of circumdatin A was finally established to be the same as reported for asperloxin A (**2**) [17]. The first oxepine-containing alkaloid with a phenylalanine residue brevianamide L (**9**) containing a 12-hydroxyl dihydro-oxepine ring, together with brevianamides O and P (**10**–**11**), was isolated from the solid-state fermented rice culture of *Aspergillus versicolor* (AS 3.4186) [8,9]. Oxepinamide D (**12**) and oxepinamides E–G (**13**–**15**), containing a 12-oxygenated-oxepine ring, were isolated from *Aspergillus puniceus* F02Z-1744 grown on solid media containing rice and soybean [24]. Protuboxepins A (**16**) and B (**17**) were isolated from the marine-derived fungus *Aspergillus* sp. SF-5044, whose 28S rRNA gene (Genbank accession number FJ935999) showed a high-sequence identity of 99.64% with that of *Aspergillus protuberus* (FJ176897) [25]. Circumdatin L (**18**) was isolated from the solid rice culture of *Aspergillus westerdijkiae* DFFSCS013 [26]. Dihydrocinereain (**19**) with cinereain (**1**) was characterized from a marine strain of *Aspergillus carneus* KMM 4638 grown on modified rice medium with seawater [27]. Varioxepine A (**20**) bearing a unique oxa-cage was isolated from the marine algal-derived fungus *Paecilomyces variotii* EN-291 fermented in potato dextrose broth medium [28]. Varioloids A and B (**21**–**22**) with protuboxepin B (**17**) were also isolated from *Paecilomyces variotii* EN-291 fermented in the same condition [10]. Versicoloids A and B (**23**–**24**) were isolated from the deep-sea-derived fungus *Aspergillus versicolor* SCSIO 05879 grown in liquid medium containing starch and polypeptone [29]. Versicomide D (**25**) was isolated from *Aspergillus versicolor* XZ-4 fermented in liquid medium with seawater [30]. Protuboxepins C and D (**26**–**27**) were isolated from the sponge-derived fungus *Aspergillus* sp. SCSIO XWS02F40, which was found to belong to a clade related to *Aspergillus austroafricanus* NRRL 233 with an identity of 99.4% using ITS1-5.8S-ITS2 sequence region [31,32]. Chrysopiperazines A and B (**28**–**29**) with versicoloids A and B (**23**–**24**) were obtained from a gorgonian-derived *Penicillium chrysogenum* strain (CHNSCLM-0019), and their absolute configurations were completely solved by NOESY, Marfey’s reaction, and electronic circular dichroism (ECD) and vibrational circular dichroism (VCD) methods [33]. Protuboxepins F (**30**) and G (**31**) were isolated from the marine sponge-derived fungus *Aspergillus versicolor* SCSIO 41016 grown on solid rice media with artificial sea salt [34]. Oxepinamides H−K (**32**–**35**) were isolated from a deep-sea-derived *Aspergillus puniceus* SCSIO z021 fermented in liquid medium with sea salt [35].

### 2.2. Bioactivities

#### 2.2.1. Plant Growth Regulation

Cinereain (**1**), the first OPK peptide, could significantly inhibit the growth of etiolated wheat coleoptiles (*p* < 0.01) at 10^−4^ and 10^−3^ M and cause mild necrosis and chlorosis in corn, but it did not have any effect on intact greenhouse-grown bean and tobacco plants [6].

#### 2.2.2. Anti-Inflammatory Activity

In a topical resiniferatoxin (RTX)-induced mouse ear edema assay, oxepinamide A (**4**) showed good topical anti-inflammatory activity with 82% inhibition of edema at the standard testing dose of 50 µg per ear [20].

#### 2.2.3. Antifungal Activity

Oxepinamides A–C (**4**–**6**) showed no antifungal activity toward *Candida albicans* in a broth micro-dilution assay [20]. Janoxepin (**7**) showed no antifungal activity in an in-house disc diffusion assay [21]. Brevianamide L (**9**) showed no inhibitory activity against *Candida albicans* at a concentration of 100 µg/mL [8]. However, varioxepine A (**20**) and varioloids A and B (**21**–**22**) exhibited activity against the plant-pathogenic fungus *Fusarium graminearum* with MIC values of 4, 8 µg/ml, respectively [10,28]. Versicoloids A and B (**23**–**24**) exhibited antifungal activities against the three phytopathogenic fungi *Colletotrichum acutatum*, *Magnaporthe oryzae*, and *Fusarium oxysporum*, both with MICs of 1.6, 128, and 64 μg/mL. Their activity against *Colletotrichum acutatum* was even stronger than the positive control cycloheximide (MIC of 6.4 μg/mL), and they could be regarded as candidate agrochemical antifungal agents [29]. Chrysopiperazine A (**28**) did not show activity against *Candida albicans* at the concentration of 50 µM [33]. Oxepinamides H–K (**32**–**35**) showed low percent inhibition (< 50%) against the four phytopathogenic fungi—*Curvularia australiensis*, *Colletotrichum gloeosporioides*, *Fusarium oxysporum*, and *Pyricularia oryzae*—at a concentration around 0.6 mM [35].

#### 2.2.4. Cytotoxicity

Oxepinamides A–C (**4**–**6**) showed no significant activity against any cell line in the National Cancer Institute´s 60 cell-line panel [20]. Circumdatin B (**8**) was also tested in the NCI´s 60 cancer cell line panel and did not show activity either [22]. Neither Circumdatin A (**2**) nor Circumdatin B (**8**) showed cytotoxicity against A548 lung cancer cells [23]. Brevianamides L, O, and P (**9**–**11**) showed no cytotoxicity against human breast cancer (Bre04), human lung (Lu04), or human neuroma (N04) cell lines (GI_50_ > 10 µg/mL) [8,9]. Protuboxepin A (**16**) showed weak inhibitory activity against human acute promyelocytic leukemia cells (HL-60), human breast cancer adenocarcinoma cells (MDA-MB-231), hepatocellular carcinoma cells (Hep3B), rat fibroblast cells (3Y1), and chronic myelogenous leukemia cells (K562), with IC_50_ values of 75, 130, 150, 180, and 250 μM, respectively [25]. A further in vitro study revealed that this compound could bind to α- and β-tubulin and thereby stabilize tubulin polymerization, altogether disrupting microtubule dynamics. This disruption led to chromosome misalignment and metaphase arrest, inducing apoptosis in tumor cells [37]. The compound circumdatin L (**18**) did not show cytotoxicity toward the human carcinoma A549, HL-60, K562, and MCF-7 cell lines (IC_50_ > 10 μM) [26]. Dihydrocinereain (**19**) was tested against murine ascites Ehrlich carcinoma cells but did not show activity up to 100 μM [27]. Similarly, protuboxepins C and D (**26**-**27**) showed no inhibitory activity against A549 cells with IC_50_ values of 100 and 190 μM and weak activities against HeLa cells with IC_50_ values of 61 and 114 μM [31]. Protuboxepin G (**31**) displayed moderate cytotoxic activities against three renal carcinoma cell lines (ACHN, OS-RC-2, and 786-O cells) with the IC_50_ values 27.0, 47.1, and 34.9 μM, respectively [34].

#### 2.2.5. Antibacterial Activity

In disk assays with cinereain (**1**) against *Bacillus subtilis*, *Bacillus cereus*, and *Mycobacterium thermosphactum* (Gram-positive), and *Escherichia coli* and *Citrobacter freundii* (Gram-negative), no effects were observed in concentrations up to 500 μg per disk [6]. Janoxepin (**7**) showed no antibacterial activity in an in-house agar overlay assay [21]. Circumdatins A (**2**) and B (**8**) were subjected to an inhibitory test against Methicillin-resistant *Staphylococcus aureus* (MRSA), but no activities were observed [23]. Brevianamide L (**9**) showed no inhibitory activity against *Escherichia coli*, *Staphylococcus aureus*, and *Pseudomonas aeruginosa*, at a concentration of 100 µg/mL [8]. Varioxepine A (**20**) and Varioloids A and B (**21**–**22**) showed promising antibacterial activities against *Micrococcus luteus*, *Staphylococcus aureus*, *Escherichia coli*, and the aquacultural bacteria *Aeromonas hydrophila*, *Vibrio anguillarum*, *Vibro harveyi* and *Vibro parahaemolyticus*, with MIC values ranging from 16 to 64 μg/ml [10,28]. Versicomide D (**25**) was applied to three pathogenic bacteria (*E. coli*, *S. aureus* and *B. subtilis*), but no MIC values were reported. Chrysopiperazine A (**28**) did not show activity against *Escherichia coli*, *Staphylococcus aureus*, *Pseudomonas aeruginosa*, *Photobacterium halotolerans*, and *Enterobacter cloacae*, at the concentration of 50 µM [33].

#### 2.2.6. Anti-Plasmodial Activity

Janoxepin (**7**) exhibited antiplasmodial activity against the malaria parasite *Plasmodium falciparum* 3D7 in the radioisotope assay with IC_50_ lower than 28 mg/mL [21].

#### 2.2.7. Transcriptional Activation

Selective transactivation effects of oxepinamides D–G (**12**–**15**) were examined, and they selectively showed moderate transcriptional activation on Liver X Receptor α (LXRα) with EC_50_ values of 10.6, 12.8, 13.6, and 12.1 µM, but no agonistic effects were observed towards other seven nuclear receptors FXRα, PPARα, PPARβ, PPARγ, RARα, RXRα, or ERα [24]. Oxepinamides H−K (**32**–**35**) later also showed the same activation effects on LXRα with EC_50_ values of 15, 15, 16, and 50 µM, respectively, but did not show inhibition activity against other seven enzymes [35].

### 2.3. Biosynthesis

The biosynthesis of OPK NRPs remains unsolved despite the fact that some biosynthetic pathway studies have been performed on similar quinazolinone alkaloids [38,39,40,41]. Possible biosynthetic pathways of several OPK compounds have, however, been proposed. Janoxepin (**7**) was suggested to be derived from the condensation of anthranilic acid with a diketopiperazine ring formed between two leucine residues, followed by oxidation of the benzoyl derivative to give the oxepine derivative [21]. Similarly, oxepinamide D (**12**) was proposed to be biosynthesized by the condensation of a diketopiperazine with an anthranilic acid and subsequent oxidation of the benzene ring to form an arene oxide, which was opened through a thermal 6π electrocyclic ring-opening process. Oxepinamides E–G (**13**–**15**) were formed by dehydration on the 2,5-diketo ring, followed by the addition of water between C-6 and C-12 [24]. Circumdatins A (**2**) and B (**8**) were proposed to be biosynthesized by oxidation of circumdatins H and J to form a benzene oxide, where a retro-pericyclic reaction (benzene oxide–oxepine tautomerism) took place to produce the final products [23,42]. Similar to janoxepin (**7**), the backbone of varioxepine A (**20**) has also been proposed to be from the condensation of anthranilic acid with a diketopiperazine, followed by epoxidation of the benzene ring to form the oxepine derivative. A series of reactions were proposed, including a second epoxidation, ring arrangement, epoxy opening, prenylation, dihydroxylation, and/or cyclization to yield the end product [28]. Protuboxepin D (**27**) was proposed to be formed by condensation of D-phenylalanine, L-isoleucine, and anthranilic acid, followed by oxidation of the benzene ring to form the oxepine derivative through an epoxy precursor and sequent oxidation at C-3 to form the hydroxyl group. Protuboxepin C (**26**) was a methylation product of protuboxepin D (**27**) [31]. A recent report proposed that additional opening and oxidation could happen on the oxepine ring, which then may undergo addition of water, cyclization, and methylation to yield unique (di/tetra)-hydropyran-pyrimidinone-ketopiperazine heterotricyclic products [34].

## 3. Discussion

**Figure 2:**
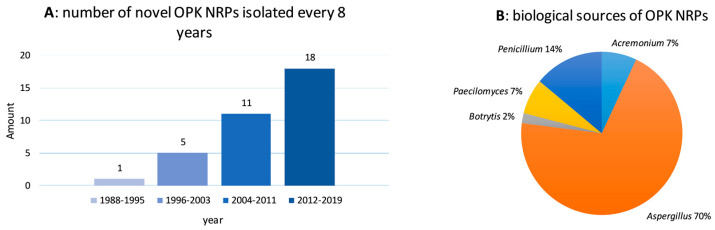
(**A**) number of novel Oxepine-Pyrimidinone-Ketopiperazine NRPs isolated every eight years, (**B**) biological sources of OPK NRPs at the genera level.

In total, thirty-five OPK compounds have currently been characterized from natural sources. The speed of novel OPK product discovery has been increasing in recent years, as over half of the currently described products were isolated during the past eight years (Figure 2A). It is quite noteworthy that all these compounds were isolated from five fungal genera. Specifically, 70% of OPK NRPs, including the rediscovered cases, were obtained from the genus *Aspergillus*, followed by genus *Penicillium* accounting for 14%, *Acremonium* 7%, *Paecilomyces* 7%, and *Botrytis* 2% (Figure 2B). Interestingly, all type C producers are from *Aspergillus* section *Circumdati*, including *A. ochraceus, A. ostianus*, and *A. westerdijkiae*, and a large proportion of type A and B compounds were obtained from different isolates of in particular the two species *A. versicolor* and *A. protuberus*, both belonging to *A. versicolor* clade in section *Nidulantes* [43,44,45]. In general, OPK compounds have been reported from species in the closely related fungal families *Aspergillaceae* (*Aspergillus, Penicillium*) and *Trichocomaceae* (*Paecilomyces*). *Botrytis cinerea* and *Acremonium* species are distantly related to *Aspergillaceae* and *Trichocomaceae*, but they were also reported to produce OPK compounds. Unfortunately, several of the reported strains have not been deposited in any culture collections affiliated to the World Federation for Culture Collections (WFCC), which is possibly why their identity has not been validated. It is also notable that even though some species reported bear the initials of a collection center, their strain number cannot be traced in the corresponding collection system. For example, *Aspergillus ochraceus* DSM 7428 cannot be found in DSMZ collection, and *Aspergillus versicolor* (AS 3.4186) cannot be traced in CGMCC collection. While the identification of *Botrytis cinerea* (ATCC 64157) can be verified, the identification of *Acremonium* (strain unavailable) was based on fatty acid methyl ester (FAME) profiles, a method which has not been authenticated for identification purposes in filamentous fungi. Genome mining of *Botrytis* and *Acremonium* species will show whether OPK compounds are taxonomically widespread or restricted to *Aspergillaceae* and *Tricocomaceae*.

Based on the number of rings and conjugation systems on the backbone, OPK NRPs were categorized into three types: A, B, and C. Type A dominating the OPK NRPs with 25 compounds shares the same 7/6/6 backbone, whereas type B OPK’s contains a larger conjugation system. Type C 7/6/7/6 backbone has one more ring than types A and B due to incorporation of a second anthranilic acid moiety, and some products even display a complex 7/6/7/6/5 ring system with an additional pyrrolidine-ring from proline. In nature, a lot of other OPK similar products have been isolated, such as the quinazolinones [7,13]. Due to their possible related biosynthetic pathways, mistakes might happen during structure elucidation [22,23]. One common issue with OPK compounds is the absolute configuration (AC) determination of α carbons and R groups of the amino acids. In many reports, NOESY, Marfey´s reaction, X-ray crystallography, and ECD methods were applied. However, one might observe a mixture of D- and L- products after the acid hydrolysis and derivation process when using Marfey´s reaction method. Hydrolysis conditions thus may need to be optimized. In the case of a chiral center at a flexible position, it can be very challenging to solve the correct configuration. Success has recently been achieved by comparing the experimental VCD spectrum with calculated data [33]. The chiral centers within the R group of the isoleucine residue in eight OPK compounds (**4**, **5**, **9**, **10**, **11**, **16**, **23**, and **24**) remain uncharacterized. The chirality also makes it confusing when referring to a structure in a publication. For example, the drawings of oxepinamide E and F (**13**–**14**) showed a 17*R* configuration (wrong) but was described as 17*S* (correct configuration by X-ray Crystallography) in the same paper [24]. Additionally, the chiral center of janoxepin (**7**) was determined as *R* configuration by Marfey´s method, but the drawing mistakenly exhibited *S* configuration [21]. Such errors also happened when the structures were drawn in different publications, like the chirality of the two α carbons of both versicoloids A and B (**23**–**24**) drawn in a recent paper [33], which displayed opposite configurations from the original structures [29]. Care should be taken to avoid making such erroneous configurational drawings. Moreover, it is also notable that both D- and L- amino acids can participate in building the OPK products based on all the characterized structures. Therefore, proposing the chirality of α carbon from a biogenetic prospect can be challenging.

A wide range of bioassays have been applied to assess the potential bioactivity of the OPK type of compounds. Though they in general seem to be inactive against human pathogenic strains of *Candia albicans*, some showed potential in treating plant-pathogenic fungi such as *Fusarium graminearum* and *Colletotrichum acutatum*. Notably, protuboxepin A (**16**) has the potential to become a new and effective anticancer drug as it displayed antiproliferative activity by disrupting microtubule dynamics through the tubulin polymerizing in tumor cells [37] despite several other reports showing that OPK compounds did not seem to be active against cancer cells. Antibacterial tests have shown that varioloids A and B (**21**–**22**) exhibited promising activities against several species, while the rest of antibacterial tests did not display antibacterial activity effects. Interestingly, oxepinamides D-G and H−K (**12**–**15**, **32**–**35**) all selectively showed transactivation effects on LXRα, which implied their potential use as novel LXR agonists in the treatment of atherosclerosis, diabetes, and Alzheimer’s disease.

**Figure 3:**
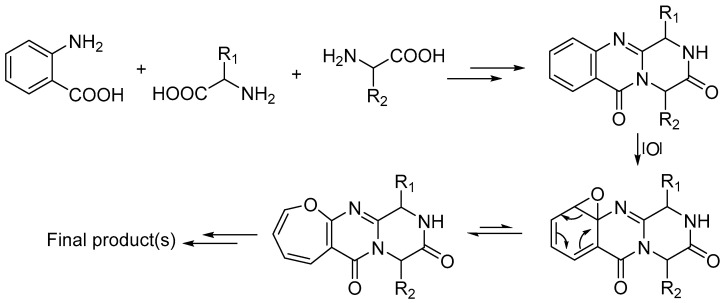
Proposed common biosynthetic steps of Oxepine-Pyrimidinone-Ketopiperazine NRPs.

Overall, this class of compounds seem to share similar biosynthetic steps to form the OPK backbone, which is likely biosynthesized by the condensation of three amino acids, including one or two anthranilic acids, to form the tricyclic core. Subsequent epoxidation on the benzene ring of the first anthranilic acid residue followed by a ring rearrangement then produces the oxepine moiety. Several successive tailoring reactions can happen before the full construction of the final product(s) (Figure 3). Based on the knowledge of the biosynthesis of fumiquinazolines, and their well documentated proposed biosynthetic pathways, we anticipate that the OPK NRPs biosynthetic gene cluster contains at least a tri-modular NRPS gene with three adenylation domains, including one or two anthranilate-activating domains, and a gene responsible for oxidizing the phenyl moiety of the anthranilic residue to form the oxepine unit [39,40]. Additionally, an epimerization domain as part of NRPS is needed to convert L-amino acids to D-amino acids in the structures with a D-amino acid residue. Other tailoring genes are also required to encode for OPK related enzymes such as anthranilate synthase, oxidoreductases, and transporters.

## 4. Conclusions

All the OPK NRPs described here were isolated from fungal sources with most compounds reported from species within the families *Aspergillaceae* (*Aspergillus, Penicillium*) and *Trichocomaceae* (*Paecilomyces*). Type A and B compounds share the same 7/6/6 backbone, with the former dominating OPK NRPs with twenty-five reported compounds, while Type C OPKs have a larger 7/6/7/6 backbone with four products reported. In general, these compounds showed promising activities against various phytopathogenic fungi and exhibited transactivation effects on LXRα. In addition, the skeleton of OPK NRPs is likely derived from the condensation of three amino acids, including one or two anthranilic acid(s), and the oxepine moiety is formed by the epoxidation of the benzene ring followed by ring arrangement. However, experimental investigation is needed to support this hypothesis. With the advance of separation skills and spectroscopic techniques, more oxepine-containing compounds are likely to be discovered. Considering that many of these compounds were reported from Aspergilli, ongoing whole genome sequencing of all species in genus *Aspergillus* will possibly set the scene for genomic driven approaches towards new OPK NRPs [46,47].

## Figures and Tables

**Table 1:** Structures and Biological sources of Type A Oxepine-Pyrimidinone-Ketopiperazine NRPs.

No.	Name	Substitution groups					Sources
		R_1_	R_2_	R_3_	R_4_	R_5_	
**1**	Cinereain	=CHCH(CH_3_)_2_, *Z*	n/a	H	CH(CH_3_)_2_	H	*Botrytis cinerea* ATCC 64157 [6] *Aspergillus carneus* KMM 4638 [27]
**4**	Oxepinamide A	CH(CH_3_)CH_2_CH_3_	OH	OCH_3_	H	CH_3_	*Acremonium* sp. [20]
**5**	Oxepinamide B	OH	CH(CH_3_)CH_2_CH_3_	OCH_3_	H	CH_3_	*Acremonium* sp. [20]
**6**	Oxepinamide C	CH_2_CH(CH_3_)_2_	OCH_3_	OCH_3_	H	CH_3_	*Acremonium* sp. [20]
**7**	Janoxepin	=CHCH(CH_3_)_2_, *Z*	n/a	H	H	CH_2_CH(CH_3_)_2_	*Aspergillus janus* IBT 22274 [21]
**10**	Brevianamide O	OH	CH(CH_3_)CH_2_CH_3_	H	Benzyl	H	*Aspergillus versicolor* (AS 3.4186) [9]
**11**	Brevianamide P	H	CH(CH_3_)CH_2_CH_3_	H	Benzyl	H	*Aspergillus versicolor* (AS 3.4186) [9]
**12**	Oxepinamide D	OH	Benzyl	H	H	CH_3_	*Aspergillus puniceus* F02Z-1744 [24]
**16**	Protuboxepin A	CH(CH_3_)CH_2_CH_3_	H	H	H	Benzyl	*Aspergillus* sp. SF-5044 [25] *Penicillium expansum* Y32 [36]
**17**	Protuboxepin B	CH(CH_3_)_2_	H	H	H	Benzyl	*Aspergillus* sp. SF-5044 [25] *Paecilomyces variotii* EN-291 [10] *Penicillium expansum* Y32 [36]
**19**	Dihydrocinereain	H	CH_2_CH(CH_3_)_2_	H	CH(CH_3_)_2_	H	*Aspergillus carneus* KMM 4638 [27]
**22**	Varioloid B	OCH_3_	CH(CH_3_)_2_	H	Benzyl	H	*Paecilomyces variotii* EN-291 [10]
**23**	Versicoloid A	H	CH(CH_3_)CH_2_CH_3_	OCH_3_	CH(CH_3_)_2_	H	*Aspergillus versicolor* SCSIO 05879 [29] *Penicillium chrysogenum* CHNSCLM-0019 [33]
**24**	Versicoloid B	OH	CH(CH_3_)CH_2_CH_3_	OCH_3_	CH(CH_3_)_2_	H	*Aspergillus versicolor* SCSIO 05879 [29] *Penicillium chrysogenum* CHNSCLM-0019 [33]
**25**	Versicomide D	CH(CH_3_)CH_2_CH_3_, 18*S*	H	OCH_3_	CH(CH_3_)_2_	H	*Aspergillus versicolor* XZ-4 [30]
**26**	Protuboxepin C	CH(CH_3_)CH_2_CH_3_, 16*S*	OCH_3_	H	H	Benzyl	*Aspergillus* sp. SCSIO XWS02F40 [31]
**27**	Protuboxepin D	CH(CH_3_)CH_2_CH_3_, 16*S*	OH	H	H	Benzyl	*Aspergillus* sp. SCSIO XWS02F40 [31]
**28**	Chryzopiperazine A	CH(CH_3_)CH_2_CH_3_, 19*S*	OCH_3_	OCH_3_	H	CH(CH_3_)_2_	*Penicillium chrysogenum* CHNSCLM-0019 [33]
**29**	Chrysopiperazine B	OCH_3_	CH(CH_3_)CH_2_CH_3_, 19*S*	OCH_3_	H	CH(CH_3_)_2_	*Penicillium chrysogenum* CHNSCLM-0019 [33]
**30**	Protuboxepin F	=CHCH(CH_3_)_2_, *Z*	n/a	H	H	Benzyl	*Aspergillus versicolor* SCSIO 41016 [34]
**31**	Protuboxepin G	=CHCH(CH_3_)_2_, *E*	n/a	H	H	Benzyl	*Aspergillus versicolor* SCSIO 41016 [34]
**32**	Oxepinamide H	OCH_3_	Benzyl	H	H	CH_3_	*Aspergillus puniceus* SCSIO z021 [35]
**33**	Oxepinamide I	Benzyl	OCH_3_	H	H	CH_3_	*Aspergillus puniceus* SCSIO z021 [35]
**34**	Oxepinamide J	Benzyl	OH	H	H	CH_3_	*Aspergillus puniceus* SCSIO z021 [35]
**35**	Oxepinamide K	=CH-Phenyl, *Z*	H	H	H	CH_3_	*Aspergillus puniceus* SCSIO z021 [35]

Note: backbone numberings follow Figure 1, and the other numberings are based on the original publications. n/a: not applicable due to double bond substitution.

**Table 2:** Structures and Biological sources of Type B Oxepine-Pyrimidinone-Ketopiperazine NRPs.

No.	Name	Substitution Groups		Sources
		R_6_	R_7_	
**9**	Brevianamide L	CH(CH_3_)CH_2_CH_3_	OH, 12*S*	*Aspergillus versicolor* (AS 3.4186) [8]
**13**	Oxepinamide E	CH(CH_3_)CH_2_CH_3_, 17*S*	OH, 12*R*	*Aspergillus puniceus* F02Z-1744 [24]
**14**	Oxepinamide F	CH(CH_3_)CH_2_CH_3_, 17*S*	OCH_3_, 12*R*	*Aspergillus puniceus* F02Z-1744 [24]
**15**	Oxepinamide G	CH(CH_3_)_2_	OCH_3_, 12*R*	*Aspergillus puniceus* F02Z-1744 [24]
**20**	Varioxepine A	CH(CH_3_)_2_	See Figure 1	*Paecilomyces variotii* EN-291 [28]
**21**	Varioloid A	CH(CH_3_)_2_	O(CH_2_)COCH(CH_3_)_2_, 12*R*	*Paecilomyces variotii* EN-291 [10]

**Table 3:** Structures and Biological sources of Type C Oxepine-Pyrimidinone-Ketopiperazine NRPs.

No.	Name	Scaffold	Sources
**2**	Asperloxin A (Circumdatin A)	7/6/7/6/5, Figure 1	*Aspergillus ochraceus* DSM 7428 [17] *Aspergillus ochraceus* IBT 12704 [22] *Aspergillus ostianus* 01F313 [23]
**3**	Asperloxin B	7/6/7/6/5, Figure 1	*Aspergillus ochraceus* DSM 7428 [18]
**8**	Circumdatin B	7/6/7/6/5, Figure 1	*Aspergillus ochraceus* IBT 12704 [22] *Aspergillus ostianus* 01F313 [23]
**18**	Circumdatin L	7/6/7/6, Figure 1	*Aspergillus westerdijkiae* DFFSC S013 [26]

## References

[1] Schwarzer D., Finking R., Marahiel M.A. (2003). Nonribosomal peptides: From genes to products. Nat. Prod. Rep..

[2] Cohen D.J., Loertscher R., Rubin M.F., Tilney N.L., Carpenter C.B., Strom T.B. (1984). Cyclosporine: A new immunosuppressive agent for organ transplantation. Ann. Intern. Med..

[3] Raja A., LaBonte J., Lebbos J., Kirkpatrick P. (2003). Fresh from the pipeline: Daptomycin. Nat. Rev. Drug Discov..

[4] Winn M., Fyans J.K., Zhuo Y., Micklefield J. (2016). Recent advances in engineering nonribosomal peptide assembly lines. Nat. Prod. Rep..

[5] Niquille D.L., Hansen D.A., Mori T., Fercher D., Kries H., Hilvert D. (2018). Nonribosomal biosynthesis of backbone-modified peptides. Nat. Chem..

[6] Cutler H.G., Springer J.P., Arrendale R.F., Arison B.H., Cole P.D., Roberts R.G. (1988). Cinereain: A novel metabolite with plant growth regulating properties from *Botrytis cinerea*. Agric. Biol. Chem..

[7] He D., Wang M., Zhao S., Shu Y., Zeng H., Xiao C., Lu C., Liu Y. (2017). Pharmaceutical prospects of naturally occurring quinazolinone and its derivatives. Fitoterapia.

[8] Li G.Y., Yang T., Luo Y.G., Chen X.Z., Fang D.M., Zhang G.L. (2009). Brevianamide J, a New indole alkaloid dimer from fungus *Aspergillus versicolor*. Org. Lett..

[9] Li G., Li L., Yang T., Chen X., Fang D. (2010). Four New Alkaloids, Brevianamides O – R, from the fungus *Aspergillus versicolor*. Helv. Chim. Acta.

[10] Zhang P., Li X.M., Wang J.N., Wang B.G. (2015). Oxepine-containing diketopiperazine alkaloids from the algal-derived endophytic fungus *Paecilomyces variotii* EN-291. Helv. Chim. Acta.

[11] Kshirsagar U.A. (2015). Recent developments in the chemistry of quinazolinone alkaloids. Org. Biomol. Chem..

[12] Hameed A., Al-Rashida M., Uroos M., Ali S.A., Arshia, Ishtiaq M., Khan K.M. (2018). Quinazoline and quinazolinone as important medicinal scaffolds: A comparative patent review (2011–2016). Expert Opin. Ther. Pat..

[13] Resende D.I.S.P., Boonpothong P., Sousa E., Kijjoa A., Pinto M.M.M. (2019). Chemistry of the fumiquinazolines and structurally related alkaloids. Nat. Prod. Rep..

[14] Huang R.M., Yi X.X., Zhou Y., Su X., Peng Y., Gao C.H. (2014). An update on 2,5-Diketopiperazines from marine organisms. Mar. Drugs.

[15] Doveston R.G., Steendam R., Jones S., Taylor R.J.K. (2012). Total synthesis of an oxepine natural product, (±)-janoxepin. Org. Lett..

[16] Doveston R.G., Taylor R.J.K. (2012). An expedient synthesis of the proposed biosynthetic precursor of the oxepine natural product, janoxepin. Tetrahedron Lett..

[17] Fuchser J. (1996). Beeinflussung der Sekundarstoffbildung bei *Aspergillus ochraceus* durch Variation der Kulturbedingungen sowie Isolierung, Strukturaufklarung und Biosynthese der neuen Naturstoffe. Ph. D. Thesis.

[18] Michael B. (1999). Pyrralsäuren, eine neue Klasse pyrrolmaskierter Aminosäuren aus *Nocardia* sp. und neue Sekundärmetabolite aus *Aspergillus ochraceus*. Ph. D. Thesis.

[19] Bode H.B., Bethe B., Höfs R., Zeeck A. (2002). Big effects from small changes: Possible ways to explore nature’s chemical diversity. ChemBioChem.

[20] Belofsky G.N., Anguera M., Jensen P.R., Fenical W., Köck M. (2000). Oxepinamides A-C and fumiquinazolines H-I: Bioactive metabolites from a marine isolate of a fungus of the genus *Acremonium*. Chem. Eur. J..

[21] Sprogøe K., Manniche S., Larsen T.O., Christophersen C. (2005). Janoxepin and brevicompanine B: Antiplasmodial metabolites from the fungus *Aspergillus janus*. Tetrahedron.

[22] Rahbæk L., Breinholt J., Frisvad J.C., Christophersen C. (1999). Circumdatin A, B, and C: Three new benzodiazepine alkaloids isolated from a culture of the fungus *Aspergillus ochraceus*. J. Org. Chem..

[23] Ookura R., Kito K., Ooi T., Namikoshi M., Kusumi T. (2008). Structure revision of circumdatins A and B, benzodiazepine alkaloids produced by marine fungus *Aspergillus ostianus*, by X-ray crystallography. J. Org. Chem..

[24] Lu X.H., Shi Q.W., Zheng Z.H., Ke A.B., Zhang H., Huo C.H., Ma Y., Ren X., Li Y.Y., Lin J. (2011). Oxepinamides: Novel liver X receptor agonists from aspergillus puniceus. Eur. J. Org. Chem..

[25] Lee S.U., Asami Y., Lee D., Jang J.H., Ahn J.S., Oh H. (2011). Protuboxepins A and B and protubonines A and B from the marine-derived fungus *Aspergillus sp*. SF-5044. J. Nat. Prod..

[26] Peng J., Zhang X.Y., Tu Z.C., Xu X.Y., Qi S.H. (2013). Alkaloids from the deep-sea-derived fungus *Aspergillus westerdijkiae* DFFSCS013. J. Nat. Prod..

[27] Zhuravleva O.I., Afiyatullov S.S., Yurchenko E.A., Denisenko V.A., Kirichuk N.N., Dmitrenok P.S. (2013). New metabolites from the algal associated marine-derived fungus *Aspergillus carneus*. Nat. Prod. Commun..

[28] Zhang P., Mandi A., Li X.M., Du F.Y., Wang J.N., Li X., Kurtan T., Wang B.G. (2014). Varioxepine a, a 3*H*-oxepine-containing alkaloid with a new oxa-cage from the marine algal-derived endophytic fungus *Paecilomyces variotii*. Org. Lett..

[29] Wang J., He W., Huang X., Tian X., Liao S., Yang B., Wang F., Zhou X., Liu Y. (2016). Antifungal New oxepine-containing alkaloids and xanthones from the deep-sea-derived fungus *Aspergillus versicolor* SCSIO 05879. J. Agric. Food Chem..

[30] Pan C., Shi Y., Chen X., Chen C.T.A., Tao X., Wu B. (2017). New compounds from a hydrothermal vent crab-associated fungus *Aspergillus versicolor* XZ-4. Org. Biomol. Chem..

[31] Tian Y.Q., Lin S.N., Zhou H., Lin S.T., Wang S.Y., Liu Y.H. (2018). Protuboxepin C and protuboxepin D from the sponge-derived fungus *Aspergillus* sp. SCSIO XWS02F40. Nat. Prod. Res..

[32] Tian Y.Q., Lin X.P., Wang Z., Zhou X.F., Qin X.C., Kaliyaperumal K., Zhang T.Y., Tu Z.C., Liu Y. (2016). Asteltoxins with antiviral activities from the marine sponge-derived fungus *Aspergillus* 308 sp. SCSIO XWS02F40. Molecules.

[33] Xu W.F., Mao N., Xue X.J., Qi Y.X., Wei M.Y., Wang C.Y., Shao C.L. (2019). Structures and absolute configurations of diketopiperazine alkaloids chrysopiperazines A-C from the gorgonian-derived *Penicillium chrysogenum* fungus. Mar. Drugs.

[34] Luo X., Chen C., Tao H., Lin X., Yang B., Zhou X., Liu Y. (2019). Structurally diverse diketopiperazine alkaloids from the marine-derived fungus: *Aspergillus versicolor* SCSIO 41016. Org. Chem. Front..

[35] Liang X., Zhang X., Lu X., Zheng Z., Ma X., Qi S. (2019). Diketopiperazine-type alkaloids from a deep-sea-derived *Aspergillus puniceus* fungus and their effects on Liver X Receptor α. J. Nat. Prod..

[36] Fan Y.Q., Li P.H., Chao Y.X., Chen H., Du N., He Q.X., Liu K.C. (2015). Alkaloids with cardiovascular effects from the marine-derived fungus *Penicillium expansum* Y32. Mar. Drugs.

[37] Asami Y., Jang J.H., Soung N.K., He L., Moon D.O., Kim J.W., Oh H., Muroi M., Osada H., Kim B.Y. (2012). Protuboxepin A, a marine fungal metabolite, inducing metaphase arrest and chromosomal misalignment in tumor cells. Bioorganic Med. Chem..

[38] Ames B.D., Walsh C.T. (2010). Anthranilate-activating modules from fungal nonribosomal peptide assembly lines. Biochemistry.

[39] Ames B.D., Liu X., Walsh C.T. (2010). Enzymatic processing of fumiquinazoline F: A tandem oxidative-acylation strategy for the generation of multicyclic scaffolds in fungal indole alkaloid biosynthesis. Biochemistry.

[40] Gao X., Chooi Y.H., Ames B.D., Wang P., Walsh C.T., Tang Y. (2011). Fungal indole alkaloid biosynthesis: Genetic and biochemical investigation of the tryptoquialanine pathway in penicillium aethiopicum. J. Am. Chem. Soc..

[41] Yan D., Chen Q., Gao J., Bai J., Liu B., Zhang Y., Zhang L., Zhang C., Zou Y., Hu Y. (2019). Complexity and diversity generation in the biosynthesis of fumiquinazoline-related peptidyl alkaloids. Org. Lett..

[42] Henderson A.P., Mutlu E., Leclercq A., Bleasdale C., Clegg W., Henderson R.A., Golding B.T. (2002). Trapping of benzene oxide-oxepin and methyl-substituted derivatives with 4-phenyl- and 4-pentafluorophenyl-1,2,4-triazoline-3,5-dione. Chem. Commun..

[43] Samson R.A., Houbraken J.A.M.P., Kuijpers A.F.A., Frank J.M., Frisvad J.C. (2004). New ochratoxin A or sclerotium producing species in *Aspergillus* section *Circumdati*. Stud. Mycol..

[44] Chen A.J., Frisvad J.C., Sun B.D., Varga J., Kocsubé S., Dijksterhuis J., Kim D.H., Hong S.B., Houbraken J., Samson R.A. (2016). *Aspergillus* section *Nidulantes* (formerly *Emericella*): Polyphasic taxonomy, chemistry and biology. Stud. Mycol..

[45] Takahashi C., Matsushita T., Doi M., Minoura K., Shingu T., Kumeda Y., Numata A. (1995). Fumiquinazolines A-G, novel metabolites of a fungus separated from a *Pseudolabrus* marine fish. J. Chem. Soc. Perkin Trans. 1.

[46] Vesth T.C., Nybo J.L., Theobald S., Frisvad J.C., Larsen T.O., Nielsen K.F., Hoof J.B., Brandl J., Salamov A., Riley R. (2018). Investigation of inter- and intraspecies variation through genome sequencing of *Aspergillus* section *Nigri*. Nat. Genet..

[47] Kjærbølling I., Vesth T., Frisvad J.C., Nybo J.L., Theobald S., Kildgaard S., Petersen T.I., Kuo A., Sato A., Lyhne E.K. (2020). A comparative genomics study of 23 *Aspergillus* species from section *Flavi*. Nat. Commun..

